# Land-use shapes the composition and stability of soil water-stable aggregates in a plateau agro-pastoral ecotone

**DOI:** 10.7717/peerj.20643

**Published:** 2026-01-30

**Authors:** Ling Bai, Wei Wang, Yujuan Mo, Xubin Zhang, Jianchao Fu, Longfei Shu, Zining Yue, Peng Liu, Jian Hao, Xiang Liu, Deming Zhang

**Affiliations:** 1 Xining Center of Natural Resources Comprehensive Survey, China Geological Survey, Xining, China; 2 State Key Laboratory of Plateau Ecology and Agriculture, Qinghai University, Xining, China

**Keywords:** Soil aggregate, Land-use, Tibetan Plateau, Agro-pastoral transition zone

## Abstract

**Background:**

The northeastern Tibetan Plateau is a typical agro-pastoral ecotone that experiences frequent land-use changes. Maintaining stable soil aggregates in this region is key to protecting the environment and supporting food production on the Tibetan Plateau. Nevertheless, comparative data on soil aggregate composition and stability across diverse land-use types and soil depths in this region are currently limited.

**Methods:**

Herein, soil samples from 0–20 cm and 20–40 cm depths were gathered at 52 sites spanning four dominant land-use types in the northeastern Tibetan Plateau: grassland (GL), cropland (CL), orchard (OC), and planted forest (PF). The composition and stability of soil aggregates were assessed using the wet sieving method.

**Results:**

The results exhibited that silt and clay particles (SC, <0.053 mm) were the predominant aggregate fraction across all land-use types, followed by microaggregates (MIA, 0.053–0.25 mm), while small macroaggregates (SMA, 0.25–2 mm) and large macroaggregates (LMA, >2 mm) had relatively low mass proportions. Aggregate stability in the 0–20 cm layer ranked PF > GL > CL > OC, with PF and GL significantly more stable than OC. In the 20–40 cm layer, the highest aggregate stability was found in GL. Except for PF, aggregate stability varied little between different soil layers for other land-use types. Aggregate stability is positively associated with the contents of soil organic carbon, total nitrogen, and alkali-hydrolyzable nitrogen, underscoring the critical role of soil organic matter in regulating soil aggregation.

**Conclusions:**

The findings suggest that land-use type is a key determinant of soil structure in the northeastern Tibetan Plateau.

## Introduction

As the fundamental building blocks of soil, soil aggregates are key factors affecting soil aeration, permeability, and fertility (Yudina & Kuzyakov, 2023). Soil particles can be bound together to form soil aggregates through the action of cementing agents, including roots, hyphae, the secretions of roots and microorganisms, as well as metal oxides (Amézketa, 1999). Soil aggregates play a vital role in mediating water and gas exchange processes, enhancing soil structure to improve soil erosion resistance, and providing essential habitats for soil microorganisms (Nciizah & Wakindiki, 2015; Bach et al., 2018; Kravchenko et al., 2019). Additionally, soil aggregates can reduce microbial decomposition of soil organic carbon (SOC) by forming physical barriers (Blanco-Canqui & Lal, 2004), making their stability a critical factor in SOC sequestration (Mustafa et al., 2020). Consequently, in the backdrop of worldwide land degradation and climate shifts, sufficient attention should be given to changes in the composition and stability of soil aggregates.

The composition of soil aggregates is jointly affected by various factors such as vegetation type, soil organic matter (SOM) level, microbial activity, and land management practices (Tisdall, Cockroft & Uren, 1978; Six et al., 2004; Zhao et al., 2017; Edlinger et al., 2023). These factors generally vary considerably across different land-use types (Beillouin et al., 2022; Nuralykyzy et al., 2023), thereby influencing the stability of soil aggregates. As an example, differences in SOM levels across land-uses lead to variations in the amount of aggregate binding agents, which in turn affect the aggregation of soil particles (Feng et al., 2021). Human activities are the primary driver of variations in soil aggregate stability across land-use types (Zhu, Shangguan & Deng, 2021; Li et al., 2023). In general, irrational human activities (*e.g.*, overgrazing and deforestation) can degrade native vegetation and diminish the proportion of soil macroaggregates (>0.25 mm) (Singh, Singh & Ghoshal, 2017; Ma, Wang & Shen, 2020). Conversely, rational land management practices (*e.g.*, no-till farming and use of organic fertilizers) contribute to maintaining soil aggregate stability (Liu et al., 2021; Tian et al., 2022). For instance, while prior studies suggest that forests generally maintain more stable soil aggregates than croplands (Chrenková et al., 2014; Dorji, Field & Odeh, 2020), Guo et al. (2020) observed no significant shifts in mean weight diameter (MWD) and geometric mean diameter (GMD) after a decade of converting cropland to forest. Similarly, research conducted by Li et al. (2023) in northeastern China also revealed no significant distinctions in soil aggregate stability among croplands, orchards, forests, and grasslands. These discrepancies suggest that the effects of land-use on soil aggregates may vary across regions, owing to differences in climate, soil type, and land management practice (Edlinger et al., 2023).

Agro-pastoral ecotone serves as a transitional region between the distinct ecosystems of agriculture and animal husbandry, typically situated near the boundary between semi-arid and semi-humid regions (Liu et al., 2025). The integration of agricultural and pastoral production results in diverse land-use types and frequent land-use changes in the agro-pastoral ecotone (Liu et al., 2020; Liu et al., 2024). Evidence from previous studies indicates that land-use practices have a substantial impact on the composition and stability of soil aggregates in the agro-pastoral ecotone, thereby influencing soil structure and function (Yao et al., 2019; Okolo et al., 2020; Zhang et al., 2023). For example, converting grasslands into croplands generally reduces the stability of soil aggregates, a consequence associated with shifts in vegetation types and the adoption of agricultural practices (Ren et al., 2022). By comparison, the conversion of croplands to grasslands or forests can enhance soil structure by promoting the formation of macroaggregates (Zhang et al., 2023). Nevertheless, there is currently no consensus on the differences in the stability of soil aggregates between grasslands and forests in the agro-pastoral ecotone (Yao et al., 2019; Ren et al., 2022). On the Loess Plateau, Yao et al. (2019) reported that forests exhibited greater stability of soil water-stable aggregates compared to grasslands. Conversely, Zhu, Shangguan & Deng (2017) observed that natural restored grasslands demonstrated higher stability of soil water-stable aggregates than Chinese red pine forests after 15 years of restoration. Furthermore, the differences in soil aggregate stability among various land-use types exhibit distinct patterns across different soil layers in the agro-pastoral ecotone (Zhu, Shangguan & Deng, 2021; Zhang et al., 2023; Bai, Liu & Yuan, 2025). Some studies found that variations in soil aggregate across different land-use types are predominantly observed in the topsoil (Kalhoro et al., 2017; Zhang et al., 2023), while other evidence suggests that land-use types exert a more significant influence on soil aggregates in deeper soil layers (Zhu, Shangguan & Deng, 2021). Although research findings remain inconsistent, there is growing consensus that land-use type plays a key role in shaping the spatial distribution of soil aggregates in the agro-pastoral ecotone.

The northeastern Tibetan Plateau represents a typical agro-pastoral ecotone, playing a crucial role in sustaining the plateau’s food security (Ma et al., 2023; Bai, Liu & Yuan, 2025). Grassland constitutes the natural vegetation type in this region, with increasing portions being transformed into cropland and orchards (Zhang et al., 2022; Ma et al., 2023). Since 2000, some degraded croplands and orchards have been converted into planted forests through the “Grain-for-Green” Program (Zhou et al., 2019). The first three land-use types are vital in the production of regional agricultural and pastoral products, while planted forests provide important ecological functions, including water conservation and climate regulation (Ma et al., 2023). However, limited studies have compared soil aggregate stability across grasslands, croplands, orchards, and planted forests in this plateau agro-pastoral ecotone, particularly in deeper soil layers. In this study, 0–20 cm and 20–40 cm soil samples were gathered from grasslands, croplands, orchards, and planted forests in the northeastern Tibetan Plateau. Separation of soil water-stable aggregate fractions was achieved through wet-sieving methodology. This investigation was designed to address three core objectives: (i) quantify the composition and stability of soil aggregates under different land-use types; (ii) examine the link between soil aggregate stability and soil physicochemical characteristics. The study hypothesized that: (i) macroaggregates would constitute the primary component of soil aggregates in planted forest soils, leading to significantly higher soil aggregate stability in planted forests than the other three land-use types; (ii) SOC would be a key factor modulating the stability of soil aggregates.

## Materials & Methods

### Study region and soil sampling

The study region is situated in the northeastern Tibetan Plateau of China, including Xining City, Haidong Prefecture, Haibei Tibetan Autonomous Prefecture, and Hainan Tibetan Autonomous Prefecture. The region exhibits a temperate semi-arid climate, characterized by an average elevation of less than 3,000 m. Mean annual temperatures range between 2.0 and 8.6 °C, while precipitation is generally low, averaging below 500 mm and dropping to around 250 mm in some areas (Yu et al., 2012). The region encompasses 4.2 × 10^5^ hectare of cropland, with a crop growing season lasting approximately 180–240 days. The predominant crops cultivated are cool-season and drought-tolerant species, including wheat (*Triticum aestivum*), highland barley (*Hordeum vulgare*), rapeseed (*Brassica napus*), and potato (*Solanum tuberosum*). According to the USDA Soil Taxonomy, the main soil types in this region are categorized as Mollisols and Aridisols (Dai et al., 2018).

**Figure 1:**
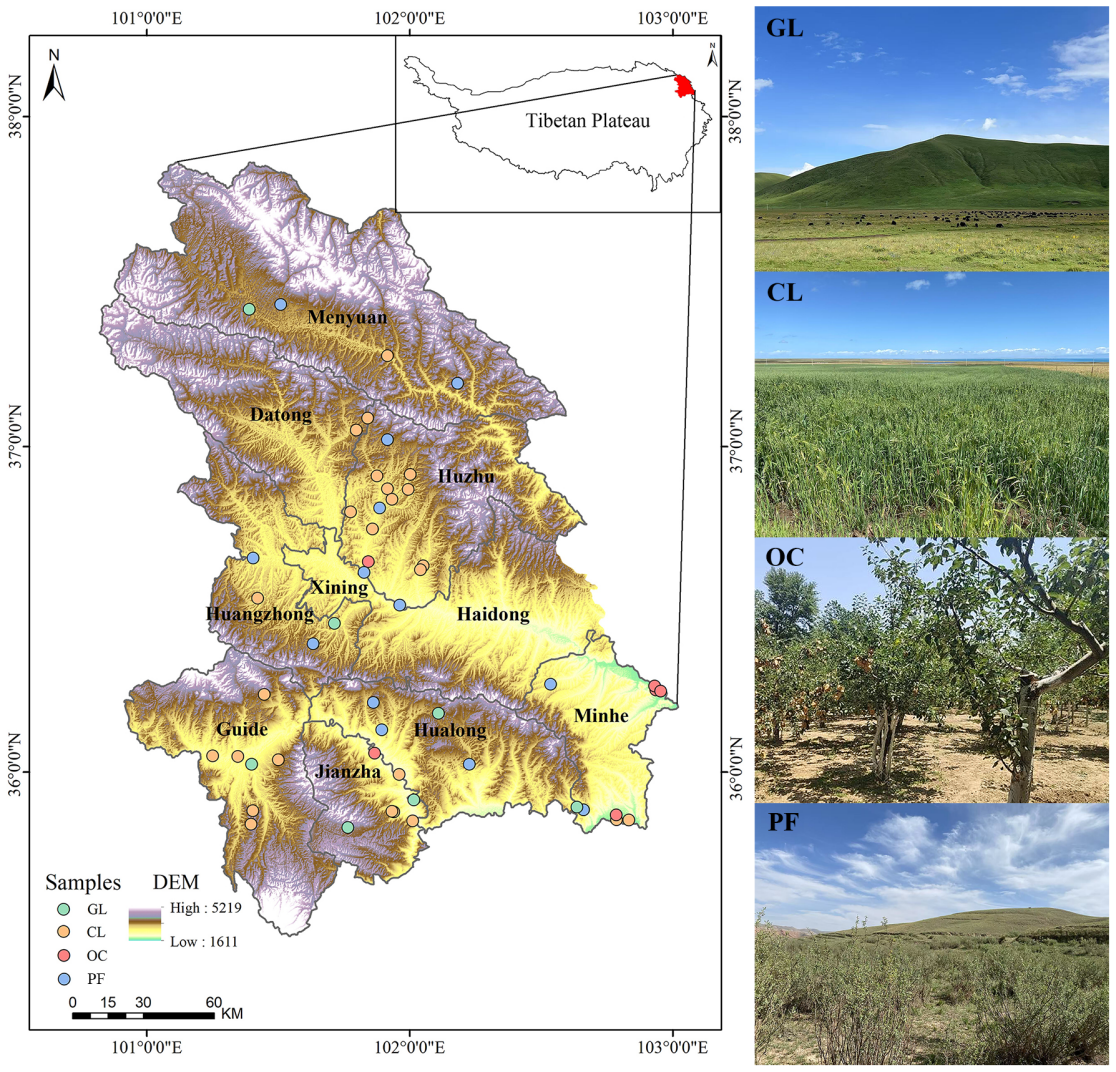
Geographical distribution of sampling sites and representative photos of each land-use type.

Soil samples were collected from April to May 2024 across 52 representative sites in the study region, encompassing four major land-use types: cropland (CL, 26 sites), grassland (GL, seven sites), orchard (OC, six sites), and forest (PF, 13 sites) (Fig. 1). To minimize the disturbance effects of short-term land-use changes on soils, sites with land-use conversion histories of less than 2 years were excluded. GL represents the native vegetation type in the study region with an average coverage of approximately 75%. CL, primarily converted from GL, has an average cultivation history of 35 years. Organic fertilizers are applied across all CL sampling sites. Transformed from either GL or CL, OC is predominantly planted with peach (*Prunus persica*), apple (*Malus pumila*) and walnut (*Juglans regia*), which have reached an average stand age of 11 years at the time of sampling. PF established under China’s “Grain-for-Green” Program primarily consists of shrubs such as *Hippophae rhamnoides* and *Caragana korshinskii*, with some sites also containing *Juniperus chinensis* and *Populus*. The average stand age across these sites is approximately 7 years. At each sampling site, three 1 × 1 m quadrats were established in a triangular configuration with 100 m spacing to ensure spatial representativeness. Within each quadrat, five soil samples were collected using a shovel from two depth intervals (0–20 cm and 20–40 cm). Within each quadrat, soil samples from the same soil layer were homogenized to obtain a composite sample for further analysis. Following this sampling protocol, a total of 312 composite soil samples (52 sites × three replications × two depth intervals) were finally collected.

### Sample analysis

Prior to aggregate separation, soil samples were air-dried in the shade and passed through an 8-mm sieve to remove plant debris and stones while maintaining soil aggregate structure. The distribution of soil aggregates was assessed using the wet-sieving method (Ma et al., 2025). Briefly, a 50 g of air-dried soil was placed on the uppermost sieve of a nested set (arranged in descending order: two mm, 0.25 mm, and 0.053 mm mesh sizes). The sample was saturated with distilled water for 30 min, after which the sieve set was placed into the bucket of a soil aggregate analyzer (TPF-100; Fangeng Technology Co., Ltd., Hebei, China). The oscillation time, frequency, and amplitude were set to 5 min, 30 cycles per minute, and four cm, respectively. Following wet-sieving, the water-stable aggregates collected on each sieve were precisely quantified and transferred to pre-weighed aluminum dishes, yielding three size fractions: (i) large macroaggregates (LMA, >2 mm), (ii) small macroaggregates (SMA, 0.25–2 mm), and (iii) microaggregates (MIA, 0.053–0.25 mm). The silt and clay particles (SC, <0.053 mm) were allowed to settle in the bucket for 48 h, after which the supernatant was discarded and the remaining material transferred to a pre-weighed aluminum dish. All aggregate fractions were dried in an oven at 60 °C to constant weight for dry mass determination.

The pH of soil was measured using a soil-water suspension (weight-to-volume ratio, 1:2.5) using a pH meter (PHS-3E; INESA Scientific Instrument Co., Ltd., Shanghai, China). SOC was determined using a high-frequency infrared carbon-sulfur analyzer (COREY-205, Kerui Instrument Co., Ltd., Deyang, China). Soil total nitrogen (STN) was measured using the Kjeldahl method, while alkali-hydrolyzable N (AN) was determined using the alkali-hydrolysis diffusion method. The molybdate-antimony-anticolorimetric method was employed to determine soil total phosphorus (STP). Soil available P (AP) was extracted with HCl-NH_4_F (pH < 7) or NaHCO_3_ (pH > 7), and P content in the extracts was also measured *via* molybdate-antimony-anticolorimetry (Liu et al., 2017). The particle size distribution of soil was analyzed according to NY/T 1121.3–2006 (Zhan et al., 2023).

### Calculation and statistical analysis

In this study, soil aggregate stability was quantitatively evaluated through four established metrics, including aggregate ratio (*AR*), mass proportion of macroaggregates (*R*_0.25_, %), *MWD* (mm), and *GMD* (mm), calculated as follows (Bhattacharyya et al., 2009; Dai et al., 2022; Ma et al., 2025):

(1) \begin{eqnarray*}AR& =({\omega }_{LMA}+{\omega }_{SMA})/({\omega }_{MIA}+{\omega }_{SC})\end{eqnarray*}

(2) \begin{eqnarray*}{R}_{0.25}& =({\omega }_{LMA}+{\omega }_{\mathrm{SMA}})\times 100\%\end{eqnarray*}

(3) \begin{eqnarray*}MWD& =\sum _{i=1}^{n}{\omega }_{i}{\bar {x}}_{i}\end{eqnarray*}

(4) \begin{eqnarray*}GMD=\exp \nolimits (\sum _{i=1}^{n}{\omega }_{i}\ln \nolimits \bar {{x}_{i}})\end{eqnarray*}

where *ω*_*LMA*_, *ω*_*SMA*_, *ω*_*MIA*_, and *ω*_*SC*_ denote the mass proportions (%) of LMA, SMA, MIA, and SC, respectively. Additionally, *ω*_*i*_ and $\bar {{x}_{i}}$ represent the mass proportion (%) and mean diameter (mm) of a specific aggregate size.

Before performing statistical analyses, the Shapiro–Wilk test was employed to evaluate the normality of the variables. When necessary, a log_10_ transformation was applied to achieve normality. Differences in soil properties across land-use types were analyzed using one-way ANOVA, followed by Duncan test for post hoc comparisons. Independent samples *t*-test was performed to evaluate differences in soil properties between soil layers. The effects of land-use type and soil layer, as well as their interaction on soil aggregate composition and stability were assessed using two-way ANOVA. Pearson’s correlation coefficients, random forest analysis, and partial least squares path modeling (PLS-PM) analysis were used to examine the relationships between soil aggregate stability and soil chemical properties. All statistical analyses were conducted using SPSS (Version 23.0) and R (version 4.4.1; R Core Team, 2024).

## Results

### Soil physicochemical properties

Soil pH across all land-use types was consistently above 8.0, reflecting uniformly alkaline conditions (Table 1). No significant differences in soil pH were detected among land-use types in either soil layer (*P* > 0.05). SOC and STN peaked in GL in both soil layers, significantly exceeding OC (*P* < 0.05). In the surface layer (0–20 cm), STP content ranged narrowly from 0.78 to 0.88 g kg^−1^ and did not differ significantly among land-use types (*P* > 0.05). In contrast, OC exhibited markedly higher STP content than that in GL and PF in the subsurface layer (20–40 cm). SAN content was lowest in OC in both soil layers, significantly below GL (*P* < 0.05), whereas SAP tended to be elevated in CL and OC relative to GL and PF. Particle-size distributions were uniform, following the order silt > sand > clay in each land-use type. In general, subsurface soils displayed higher pH, STP, SAP, clay, and sand contents, while surface soils were richer in the remaining properties.

**Table 1:** Soil physicochemical properties under different land-use types (Mean ± SE).

**Indices**	0–20 cm				20–40 cm			
	GL	CL	OC	PF	GL	CL	OC	PF
pH	8.15 ± 0.07Ab	8.17 ± 0.05Ab	8.09 ± 0.13Aa	8.08 ± 0.08Ab	8.39 ± 0.08Aa	8.35 ± 0.05Aa	8.34 ± 0.09Aa	8.38 ± 0.05Aa
SOC (g kg^−1^)	17.51 ± 3.93Aa	11.59 ± 0.71ABa	10.11 ± 0.82Ba	17.33 ± 2.30Aa	12.94 ± 4.23Aa	7.27 ± 0.54Bb	6.22 ± 0.81Bb	9.31 ± 1.54ABb
STN (g kg^−1^)	2.05 ± 0.42Aa	1.44 ± 0.09ABa	0.96 ± 0.09Ba	1.81 ± 0.23Aa	1.62 ± 0.38Aa	1.23 ± 0.10Aa	0.69 ± 0.10Ba	1.21 ± 0.16ABb
STP (g kg^−1^)	0.88 ± 0.04Aa	0.82 ± 0.02Ab	0.78 ± 0.05Ab	0.82 ± 0.02Ab	0.92 ± 0.04Ca	1.26 ± 0.04ABa	1.36 ± 0.09Aa	1.08 ± 0.07BCa
SAN (mg kg^−1^)	154.93 ± 38.69Aa	116.11 ± 7.64ABa	74.58 ± 6.17Ba	113.75 ± 13.70ABa	152.97 ± 35.11Aa	105.58 ± 7.39ABa	57.76 ± 9.54Ba	129.39 ± 19.55Aa
SAP (mg kg^−1^)	5.12 ± 1.48Ba	11.92 ± 1.56Ab	11.08 ± 2.42ABb	7.14 ± 1.36Ba	8.34 ± 1.68Ba	31.57 ± 4.24Aa	35.54 ± 3.82Aa	14.08 ± 3.91Ba
Clay (%)	6.26 ± 0.55Ab	5.30 ± 0.29ABb	4.32 ± 0.37Bb	5.55 ± 0.38ABb	15.57 ± 1.18Ba	18.59 ± 0.73ABa	20.02 ± 1.43Aa	17.41 ± 0.99ABa
Silt (%)	66.43 ± 2.57Ca	68.99 ± 1.19BCa	76.88 ± 1.55Aa	71.94 ± 1.42ABa	54.52 ± 2.37Bb	54.82 ± 1.19ABb	61.01 ± 2.46Ab	57.74 ± 2.21ABb
Sand (%)	27.50 ± 2.43Aa	25.94 ± 1.16ABa	18.74 ± 1.30Ca	22.42 ± 1.29BCa	30.02 ± 3.00Aa	26.17 ± 1.37Aa	18.67 ± 2.30Ba	24.95 ± 2.54ABa

**Notes.**

Note: GL, CL, OC, and PF represent grassland, cropland, orchard, and planted forest, respectively. SOC, STN, STP, SAN, and SAP represent soil organic carbon, soil total nitrogen, soil total phosphorus, soil alkaline-hydrolyzable nitrogen, and soil available phosphorus, respectively. Uppercase letters indicate significant differences among land-use types, while lowercase letters denote significant differences between soil layers (*P* 0˜.05).

### Composition of soil water-stable aggregate

**Figure 2:**
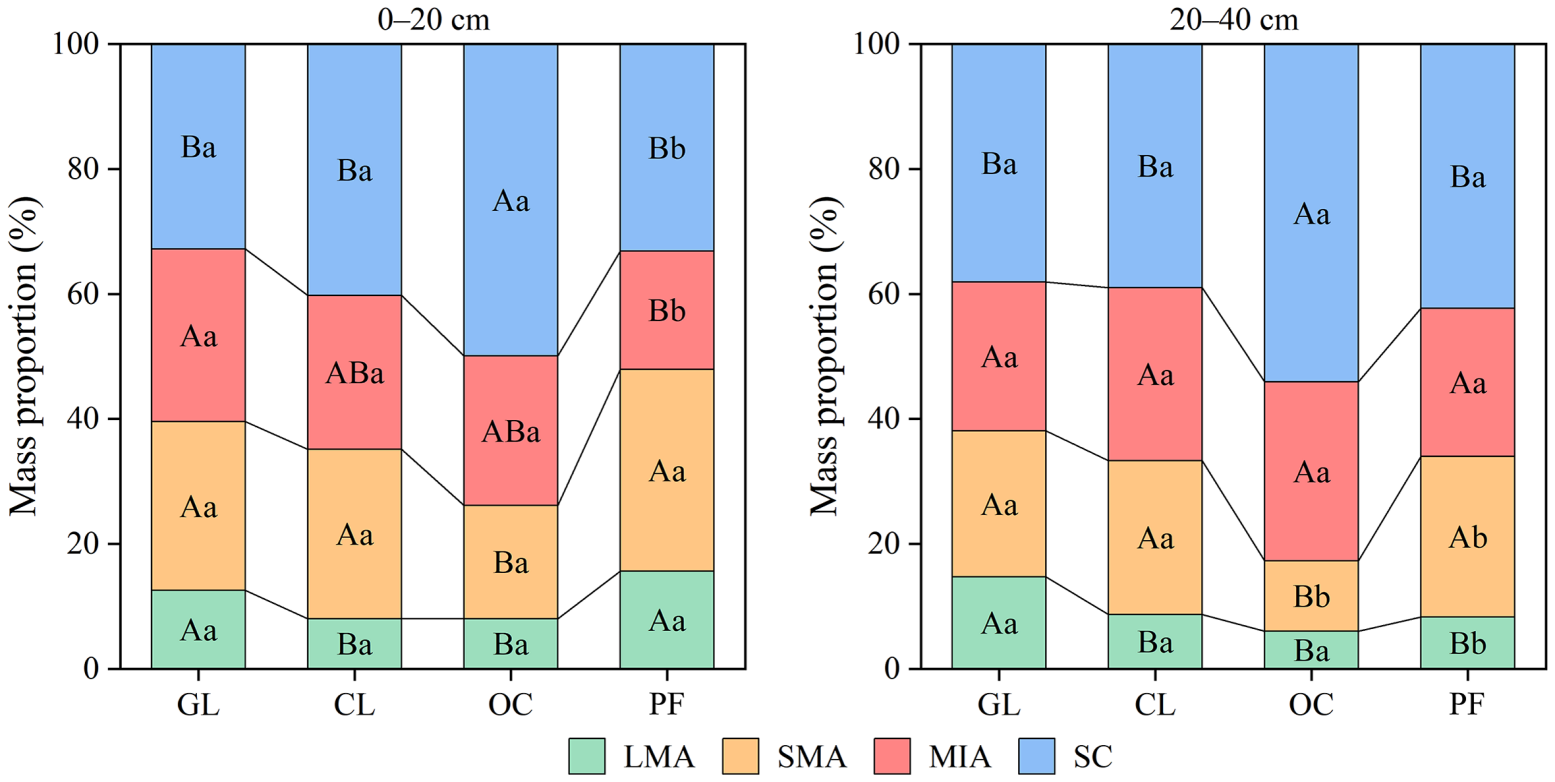
Characteristic of soil aggregate composition under different land-use types. GL, CL, OC, and PF represent grassland, cropland, orchard, and planted forest, respectively. LMA, SMA, MIA, and SC represent large macroaggregates, small macroaggregates, microaggregates, and silt and clay particles, respectively. Uppercase letters indicate significant differences among land-use types, while lowercase letters denote significant differences between soil layers (*P* < 0.05).

Across all land-use types, LMA exhibited the lowest mass proportion (7.99–15.61%), whereas SC accounted for the highest fraction (32.78–54.09%) (Fig. 2). In the surface layer, PF had significantly greater proportions of LMA and SMA than OC (*P* < 0.05), but no significant differences were observed between PF and GL or CL (*P* > 0.05). Conversely, MIA and SC reached their peak proportions in GL and OC, respectively, both significantly higher than in PF (*P* < 0.05). In the subsurface layer, CL and PF showed similar aggregate compositions (*P* > 0.05). By contrast, GL had a significantly higher proportion of LMA, and OC had a significantly higher proportion of SC than other land-use types (*P* < 0.05). Vertical variations in aggregate composition were observed in OC and PF, but not in GL or CL (*P* > 0.05). Specifically, in OC, the mass proportion of SMA was significantly higher in the surface layer than in the subsurface (*P* < 0.05), with no differences for other size classes (*P* > 0.05). In PF, the surface layer had significantly higher proportions of LMA and SMA but lower proportions of MIA and SC compared to the subsurface layer (*P* < 0.05). The results of the two-way analyis of variance (ANOVA) showed that only the mass proportion of LMA was affected by the interaction between land-use type and soil layer (*P* < 0.01) (Table 2).

**Table 2:** Two-way ANOVA shows the effects of land use type and soil layer, as well as their interaction on the composition and stability of soil aggregates (significant effects are indicated in bold).

Indices	Land-use		Soil layer		Land-use × Soil layer	
	*F*	*P*	*F*	*P*	*F*	*P*
LMA	8.006	<**0.001**	2.273	0.133	5.037	<**0.01**
SMA	11.078	<**0.001**	8.612	<**0.01**	0.621	0.602
MIA	3.845	<**0.05**	2.317	0.129	1.659	0.176
SC	7.922	<**0.001**	3.889	<**0.05**	1.825	0.143
AR	10.861	<**0.001**	8.477	<**0.01**	2.674	<**0.05**
R_0.25_	11.615	<**0.001**	8.500	<**0.01**	2.587	0.053
MWD	9.614	<**0.001**	4.662	<**0.05**	4.643	<**0.01**
GMD	10.902	<**0.001**	6.845	<**0.01**	2.817	<**0.05**

**Notes.**

Note: LMA, SMA, MIA, SC, AR, R_0.25_, MWD, and GMD represent large macroaggregates, small macroaggregates, microaggregates, silt and clay particles, aggregate ratio, mass proportion of macroaggregates, mean weight diameter, and geometric mean diameter, respectively.

### Stability of soil water-stable aggregate

**Figure 3:**
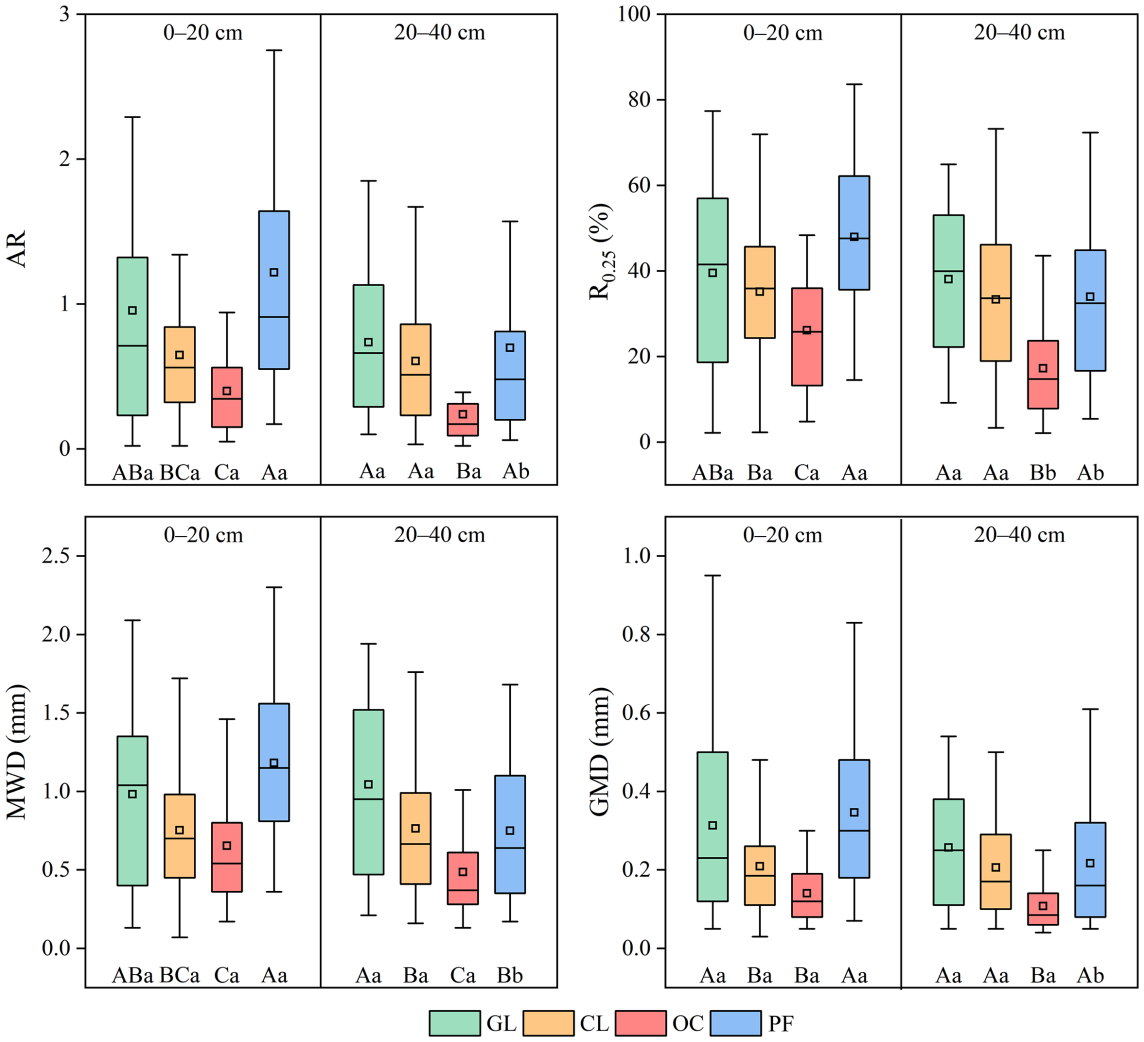
Characteristic of soil aggregate stability under different land-use types. GL, CL, OC, and PF represent grassland, cropland, orchard, and planted forest, respectively. AR, R_0.25_, MWD, and GMD represent aggregate ratio, mass proportion of macroaggregates, mean weight diameter, and geometric mean diameter, respectively. The box represents the interquartile range, while the error bars indicate maximum and minimum scores. The line inside the box indicates the median, and the square represents the mean. Uppercase letters indicate significant differences among land-use types, while lowercase letters denote significant differences between soil layers (*P* < 0.05).

In the surface layer, aggregate stability indicators ranked as follows: PF > GL > CL > OC, with PF and GL showing significantly higher stability than CL (*P* < 0.05) (Fig. 3). Conversely, in the subsurface layer, GL demonstrated the highest aggregate stability across all land-use types, with aggregate stability decreasing progressively in the order: GL > PF > CL > OC. The AR varied between 0.24 and 1.22 across land-use types and soil layers. While AR was consistently greater in the surface layer than in the subsurface layer across land-use types, the vertical difference was only significant in PF (*P* < 0.05). The R_0.25_ ranged from 26.12% to 47.95% in the surface layer and from 17.28% to 38.08% in the subsurface layer. Both OC and PF exhibited significantly greater R_0.25_ in surface soils compared to subsurface soils (*P* < 0.05). Consistent with AR, the differences in MWD and GMD between soil layers were only significant in PF (*P* < 0.05). Two-way ANOVA showed that AR, MWD, and GMD were influenced not only by land-use type and soil layer individually, but also by their interaction (*P* < 0.05) (Table 2).

### Relationships between soil physicochemical properties and soil aggregate stability

**Figure 4:**
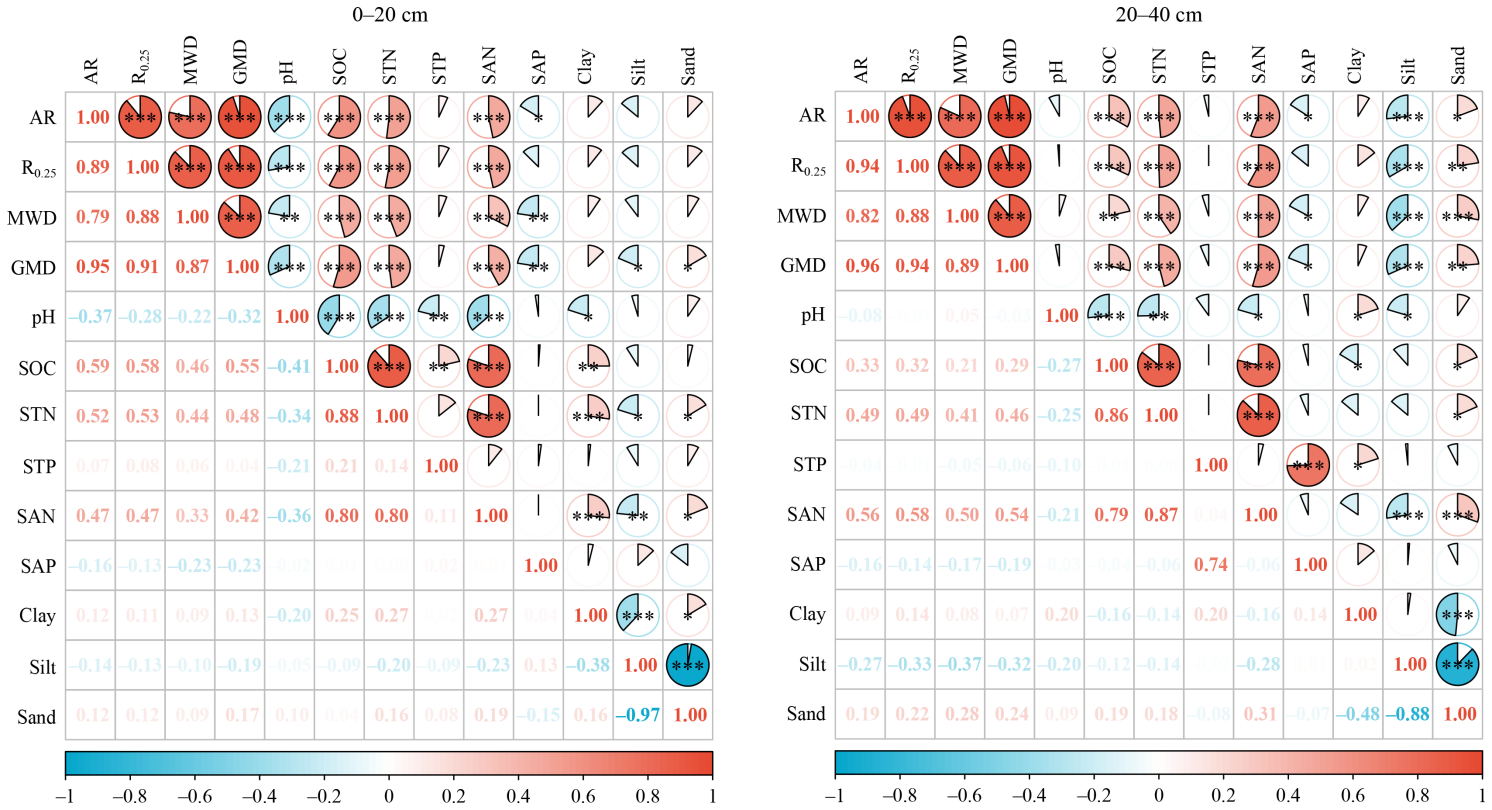
Pearson correlation analysis shows the relationships between soil physico chemical properties and soil aggregate stability. AR, R_0.25_, MWD, GMD , SOC, STN, STP, SAN and SAP represent aggregate ratio, mass proportion of macroaggregates, mean weight diameter, geometric mean diameter, soil organic carbon, soil total nitrogen, soil total phosphorus, soil alkaline-hydrolyzable nitrogen , and soil available phosphorus respectively. * *P* < 0.05; ** *P* < 0.01; *** *P* < 0.001.

**Figure 5:**
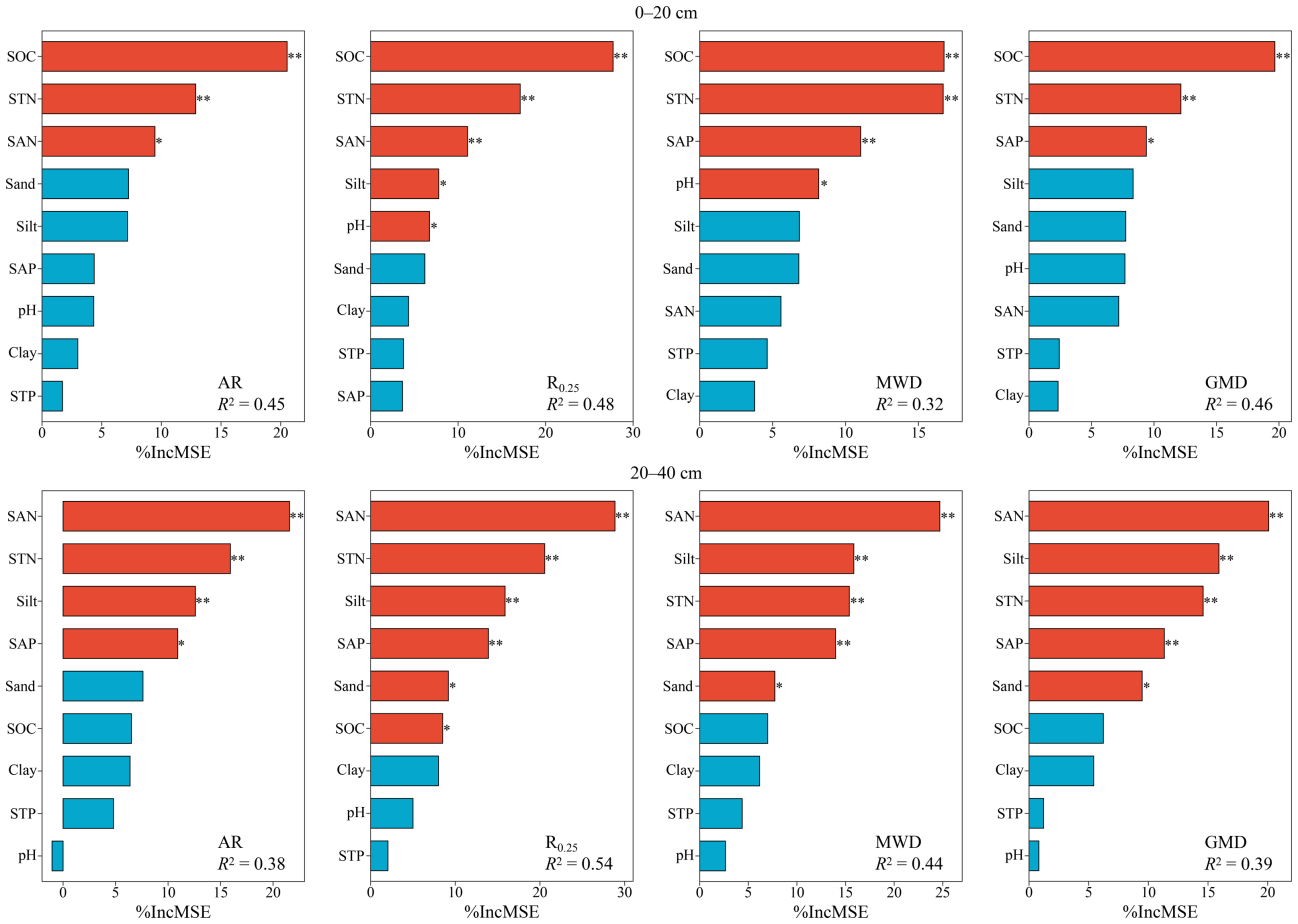
The random forest analysis reveals the relative importance of soil physicochemical properties on aggregate stability. Properties shown in red have a significant impact, whereas those in blue show negligible effects. AR, R_0.25_, MWD, GMD , SOC, STN, STP, SAN and SAP represent aggregate ratio, mass proportion of macroaggregates, mean weight diameter, geometric mean diameter, soil organic carbon, soil total nitrogen, soil total phosphorus, soil alkaline-hydrolyzable nitrogen , and soil available phosphorus respectively. * *P* < 0.05; ** *P* < 0.01.

**Figure 6:**
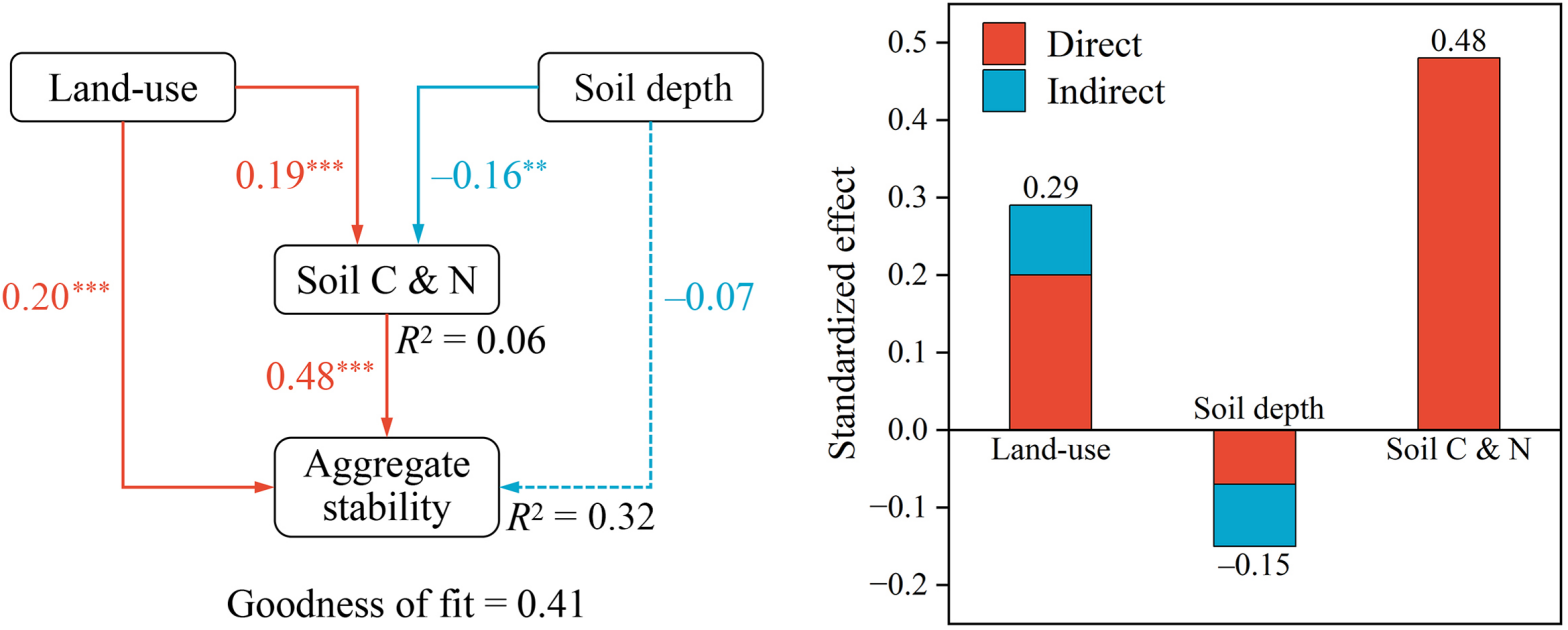
The results of the partial least squares path modeling (PLS-PM) analysis reveal the relationships among land-use types, soil depth, soil carbon and nitrogen contents (soil organic carbon, total nitrogen, and alkali-hydrolyzable nitrogen), and soil agg. Solid and dashed lines indicate significant and non-significant correlations, respectively. ** *P* < 0.01; *** *P* < 0.001.

Significant positive correlations were observed among all aggregate stability indicators in both soil layers (*P* < 0.001) (Fig. 4). Moreover, all four indicators exhibited strong positive relationships with SOC, STN, and SAN (*P* < 0.01), whereas no significant correlation was found with STP (*P* > 0.05). In the surface layer, aggregate stability was also negatively correlated with pH and SAP (*P* < 0.05). Conversely, in the subsurface layer, it showed negative correlations with SAP and silt but a positive correlation with sand (*P* < 0.05). SOC, STN, and SAP displayed strong positive correlations among themselves (*P* < 0.001), along with significant positive associations with clay in the surface layer (*P* < 0.01). In contrast, STP and SAP generally exhibited weaker correlations with the remaining variables (*P* > 0.05). Based on the results of the random forest analysis, it was observed that the stability of surface soil aggregates was predominantly influenced by SOC, STN, and SAN (Fig. 5). In contrast, the stability of subsurface soil aggregates was mainly affected by SAN, STN, SAP, silt content, and sand content. The PLS-PM analysis revealed that land-use, as well as soil C and N contents, positively influenced soil aggregate stability, with respective effects of 0.29 and 0.48 (Fig. 6). However, soil depth exhibited an inverse association with soil aggregate stability.

## Discussion

### Soil aggregate composition as influenced by land-use type

The findings revealed that SC constituted the dominant fraction across all land-use types in the plateau agro-pastoral ecotone (Fig. 2), reflecting generally poor soil aggregation. The results rejected the first hypothesis that macroaggregates would predominate in the soil aggregate composition of PF. Two primary factors likely contribute to this observed pattern: (i) water stress and temperature limitation may inhibit microbial growth (Wan et al., 2021), which is critical for aggregate formation and stabilization (Kaur et al., 2022); (ii) the sparse vegetation provides insufficient inputs of root exudates and organic residues, leading to inadequate organic binding agents for aggregate formation (Dou et al., 2020; Xiao et al., 2020). In line with the results of the present study, multiple studies in arid agroecosystems have reported similarly low proportions of macroaggregates (Mahmoodabadi & Ahmadbeigi, 2013; Wang et al., 2016). For instance, Wang et al. (2016) documented that macroaggregates accounted for <40% of total soil mass in croplands of northwestern China, while Mahmoodabadi & Ahmadbeigi (2013) found even lower values (<35%) in croplands of southeastern Iran. However, some studies pointed out that grasslands and forests in semi-arid regions typically maintained relatively high proportions of macroaggregates (>50%) (Zhu, Shangguan & Deng, 2021; Okolo et al., 2020), which contrasted with the findings in this study. Differences in vegetation characteristics, soil types, and land management regimes among individual studies likely account for the observed discrepancies. For example, all PF sites in the present study were young shrub plantations with an average stand age of 7 years, potentially restricting the development of macroaggregates.

The present study observed significant effects of land-use type on soil aggregate composition (*P* < 0.05) (Table 2), indicating that land-use conversion modified soil structure in this plateau agro-pastoral ecotone. Notably, conversion to OC could limit the formation of macroaggregates. This may be attributed to the limited understory vegetation in orchards, which reduces the inputs of plant-derived binding agents for soil aggregation. As shown in Table 1, SOC and STN contents in OC were the lowest across all four land-use types, which to some extent supported this assertion. Furthermore, infrequent tillage in OC likely results in poor aeration and water permeability, causing soil compaction that subsequently inhibits macroaggregate formation (Wei, Li & Wang, 2022). Consistent with the results of the present study, Yakupoglu et al. (2017) found that converting natural shrublands to pistachio orchards in the Gaziantep-Adıyaman Plateau of southeastern Turkey decreased the mass fraction of water-stable macroaggregates in the soil. In contrast, although significant differences in soil aggregate composition between GL and CL were observed for LMA (*P* < 0.05), the modest reductions in macroaggregates (LMA and SMA) by 11.10–12.47% after more than 35 years of cultivation were relatively smaller than those reported in other studies (Li et al., 2009; Shi et al., 2010). These findings suggest that the widespread application of organic fertilizers in CL may help mitigate substantial losses in macroaggregate proportions. The organic matter in organic fertilizers can serve as a binding agent for soil aggregates, creating favorable conditions for the formation and stabilization of soil macroaggregates (Yang et al., 2023). Ma et al. (2022) revealed that organic fertilizers outperformed inorganic fertilizers in enhancing macroaggregate formation across China’s croplands, providing partial support for the assertion. In contrast, the interaction between land-use type and soil layer only significantly affected the mass proportion of LMA (*P* < 0.01), while showing no significant effects on other aggregate size fractions (*P* > 0.05) (Table 2). This phenomenon likely arises from the intrinsic linkage between LMA formation and plant roots (Amézketa, 1999), wherein soil layer heterogeneity directly modulates root distribution patterns, consequently altering the mass fraction of LMA. These results indicate that these two factors exerted relatively independent influences on soil aggregate composition. This may be attributed to the fact that land-use type mainly alters aggregate composition through biological factors (*e.g.*, roots and soil microorganisms) (Kalhoro et al., 2017; Redmile-Gordon et al., 2020), while the influence of soil depth on soil aggregate composition is primarily driven by edaphic factors (*e.g.*, physicochemical properties) (Pan et al., 2021).

### Soil aggregate stability as influenced by land-use type

Across all land-use types in the study region, the mean R_0.25_ remained consistently below 50%, with MWD and GMD typically under 1.00 mm and 0.40 mm, respectively (Fig. 3). These findings demonstrate poor soil aggregate stability, corresponding with the low macroaggregate proportion in this plateau agro-pastoral ecotone. As mentioned above, the semi-arid climate of the study region limits the formation of macroaggregates by restricting SOM inputs. As shown in Figs. 4 and 6, aggregate stability exhibited a strong positive correlation with the contents of SOC, STN, and SAN (*P* < 0.05), indicating that SOM is a key factor governing soil aggregate stability in this region. This finding provided partial support for the second hypothesis. In general, humic substances and cellulose in SOM can chemically interact with soil mineral surfaces to form cementing agents that bind soil particles into stable aggregates (Amézketa, 1999). Simultaneously, SOM can also fill the pores within aggregates (McCarthy et al., 2008), thereby enhancing the density and structural stability of aggregates. In an agro-pastoral ecotone in northern China, Zhang et al. (2023) observed that although the water stability of soil aggregates in croplands improved after 5–25 years of vegetation restoration, the MWD remained below 1.00 mm regardless of whether the land was converted to grassland, orchard, or forest. Similarly, this study documented relatively low SOC content (<8 g kg^−1^), further indicating that SOM levels are key to aggregate stability in the agro-pastoral ecotone. Therefore, it is recommended to enhance the SOM content through appropriate management measures (*e.g.*, rational grazing, tillage, or fertilization) to improve the stability of soil structure in the study region.

In the present study, the results revealed that soil aggregate stability in the surface layer followed the order PF > GL > CL > OC, whereas in the subsurface layer, GL exhibited higher stability than PF (Fig. 3). This pattern aligns with the significant interaction between land-use type and soil layer on aggregate stability (*P* < 0.05) (Table 2). The higher aggregate stability in the surface layer of PF may be attributed to the accumulation and decomposition of litter (Hewins et al., 2017; Bougma et al., 2022; He et al., 2023). First, decomposing litter provides organic binding agents that facilitate macroaggregate formation (He et al., 2023). Second, the litter layer enhances ground coverage, mitigating raindrop impact and drying-wetting cycles that would otherwise disrupt soil aggregates (Bougma et al., 2022). Third, the decomposition of litter supplies substrates for soil microorganisms, which can promote the formation of macroaggregates (Hewins et al., 2017). In contrast, grazing results in limited litter inputs in GL, whereas agricultural practices like harvesting and tillage substantially decrease litter return in CL and OC. Empirical evidence has shown that aggregate stability can be also regulated by litter quality, which influences soil aggregation by altering the levels of microbial biomass (Laub et al., 2022). Hence, more attention should be paid in the future to the effects of both the quantity and quality of litter derived from different land-use types on soil aggregates in our study region. Consistent patterns have emerged from studies in global agro-pastoral ecotones, showing that forests generally maintain superior topsoil aggregate stability compared to other land-use types like grassland and cropland (Chrenková et al., 2014; Morlue et al., 2021; Zhang et al., 2023). In contrast, GL enhances soil aggregation primarily *via* root systems (Xiao et al., 2020), leading to greater subsurface aggregate stability than PF. However, these inferences still require further empirical validation. Consistent with findings from other agro-pastoral ecotones, the results of this study demonstrate that agricultural land-uses can reduce soil structural stability, while converting agricultural lands to PF helps to recover aggregate stability in this plateau agro-pastoral ecotone. However, given the relatively young stand age of the PF sites in this study, future investigations should evaluate how long-term afforestation influences soil aggregate composition and stability in the region.

Although our study identified land-use type and SOM levels as key factors governing soil aggregate composition and stability in this plateau agro-pastoral ecotone, the underlying mechanisms warrant further elucidation. Future research should focus on quantifying the characteristics of primary binding agents of aggregates (*e.g.*, roots, extracellular polymeric substances, and carbonates) across different land-use systems and assessing their contributions to aggregate stability. As previously highlighted, empirical studies are needed to clarify how both the quantity and quality of litter inputs influence soil aggregation. Additionally, the relationship between climatic factors and the spatial distribution of soil aggregates calls for more rigorous quantitative investigation.

## Conclusions

Using the wet-sieving method, the effects of land-use type on soil water-stable aggregates in the northeastern Tibetan Plateau were evaluated. The results revealed that LMA had the lowest mass proportion, whereas SC was the dominate fraction across all land-use types. In the surface layer, aggregate stability followed the order: PF > GL > CL > OC, with PF and GL significantly more stable than OC. In the subsurface layer, GL exhibited the highest stability. Except for PF, aggregate stability showed minimal variation across soil layers for other land-use types. The interaction between land-use type and soil depth had a weaker effect on aggregate composition than on stability. Aggregate stability exhibited a positive correlation with SOC, STN, and SAN, highlighting the pivotal role of SOM in aggregate formation. In summary, the findings of this study suggest that agricultural land-uses degrade soil structural stability, while converting agricultural lands to PF effectively restores aggregate stability in this plateau agro-pastoral ecotone. Consequently, it is recommended to enhance the SOM content of GL, CL, and OC through management practices such as rational grazing, tillage, and fertilization, thereby creating favorable conditions for the formation of macroaggregates. Additionally, degradation of PF should be prevented through measures like irrigation to maintain the stability of its soil aggregates.

## Supplemental Information

10.7717/peerj.20643/supp-1 Supplemental Information 1 The raw data used in this study

## Additional Information and Declarations

### Data Availability

The following information was supplied regarding data availability: The raw data is available in the Supplemental Files.
